# Transcriptome and WGCNA analysis revealed the molecular mechanism of drought resistance in new sugarcane varieties

**DOI:** 10.3389/fpls.2025.1687280

**Published:** 2025-11-07

**Authors:** Ziyuan Wang, Shihang Yin, Yanli Wei, Baoshan Chen, Wenlan Li

**Affiliations:** 1 State Key Laboratory for Conservation and Utilization of Subtropical Agro-Bioresources, College of Life Science and Technology, Guangxi University, Nanning, Guangxi, China; 2 Guangxi Key Laboratory for Sugarcane Biology, College of Agriculture, Guangxi University, Nanning, Guangxi, China

**Keywords:** sugarcane, drought resistance, transcriptome, WGCNA, hub genes

## Abstract

**Introduction:**

Drought stress is a major abiotic factor limiting sugarcane productivity. However, the molecular mechanisms conferring drought resistance in sugarcane are not fully elucidated, which hinders the breeding of resilient varieties.

**Methods:**

Three experimental sugarcane varieties were subjected to polyethylene glycol (PEG6000)-simulated drought stress. Subsequent transcriptomic analysis was performed by integrating second-generation (Illumina) and third-generation (PacBio) sequencing technologies. This approach yielded a comprehensive transcriptome landscape. Bioinformatics analyses included gene annotation, differential expression screening, Weighted Gene Co-expression Network Analysis (WGCNA), and network visualization using Cytoscape.

**Results:**

Sequencing generated a total transcript length of 77,930,985 bp, identifying 40,359 unique genes, with 38,791 successfully annotated. Under drought stress, the variety ZZ9 exhibited significant enrichment and upregulation of metabolic pathways related to photosynthesis, plant hormones, polysaccharide synthesis, and amino acid metabolism. Several transcription factor families, including bHLH, bZIP, ERF, NAC, MYB, and GRAS, were drought-inducible. WGCNA identified 22 co-expression modules, with the MEten module showing the highest correlation with drought response. Key hub genes within MEten included NACA1, ABA-related genes, ERA1, PER70, ATX, two superoxide dismutase genes (SODF1 and SODF2), two late embryogenesis abundant (LEA) genes, and two lipoxygenase (LOX) genes. Furthermore, Cytoscape-based analysis pinpointed the novel gene PSY1 and two additional candidates potentially involved in photosynthetic regulation during drought.

**Discussion:**

By integrating multi-platform transcriptomics and systems biology approaches, this study delineates potential molecular regulatory networks underlying drought resistance in sugarcane. The identified hub genes and pathways provide critical resources for future functional genomics studies and molecular breeding programs aimed at enhancing drought tolerance in sugarcane.

## 1 Introduction

Abiotic stresses including drought, high salinity, extreme temperatures, and excessive light impair plant growth and development, ultimately reducing crop quality and productivity (Nurrahma et al., 2024; Oyebamiji et al., 2024; Zheng et al., 2021). Field studies on crops including sugarcane, maize, wheat, soybean, and oat show that abiotic stresses can reduce yields by up to 85% (Shabbir et al., 2022; Zhang et al., 2017). In recent decades, global environmental degradation and rising temperatures have intensified the greenhouse effect, worsening drought impacts on agriculture. Additionally, many arable lands lie in arid/semi-arid regions. Increased drought frequency, combined with uneven rainfall distribution and underdeveloped irrigation, often causes prolonged drought, affecting plant morphology, photosynthesis, physiology, biochemistry, and molecular traits, severely inhibiting growth (Seleiman et al., 2021; Ravikumar et al., 2014). Drought has become one of the most critical environmental stresses limiting global agricultural productivity (Seleiman et al., 2021). Thus, studying drought stress, water use, and plant growth is vital for sustainable agriculture. Drought resistance is a complex trait governed by the interplay of genotype, developmental stage, environmental conditions, and stress characteristics including severity and duration (Zhang et al., 2023; Lim et al., 2023). However, growing evidence shows plants evolve adaptive mechanisms to cope with short/long-term drought, with stress resistance depending on genetic plasticity (Forni et al., 2016; Muchate et al., 2016), enhancing understanding of stress tolerance.

Sugarcane (*Saccharum officinarum L.*) is a vital global crop, serving as the primary source for sugar (accounting for 80% of production) (Nong et al., 2023; Ali et al., 2019) and a key feedstock for bioenergy due to its high net energy yield (Waclawovsky et al., 2010). As a perennial C4 plant, it requires abundant sunlight, warmth, water, and nutrients over its long growth cycle, confining its cultivation largely to tropical and subtropical regions. China is the world’s third-largest producer, with Guangxi province alone contributing over 60% of the national planting area. For more than a decade, the cultivar ROC22 has dominated Chinese plantations, covering approximately 70% of the total area. Global demand for sugarcane continues to rise, driven not only by sugar consumption but also by its growing importance in producing renewable fuels like ethanol (Santos et al., 2019). However, productivity faces severe challenges, particularly from drought stress (Clemente et al., 2018), which is prevalent in many rain-fed regions and significantly impairs yield and quality (Wang et al., 2015; Masoabi et al., 2018). Enhancing drought tolerance has therefore become a critical research priority. Molecular research in sugarcane was historically hampered by the lack of a reference genome, relying instead on limited expressed sequence tags (ESTs) (Carson and Botha, 2000) and transcript variants (Hoang et al., 2017). Although a reference genome was finally published in 2018 (Zhang et al., 2018; Stadermann et al., 2015), its exceptional complexity—characterized by high ploidy, extensive gene copies, and repetitive sequences—continues to pose significant challenges. Nevertheless, advances in next-generation sequencing are steadily overcoming these obstacles and facilitating deeper genetic insights.

Recent technological advances have advanced sequencing. Next-generation sequencing (NGS) technologies are widely used in biology and medicine, offering high throughput, accuracy, and low cost, but their short reads limit complete sequence assembly (Kamali and Singh, 2023). Transcriptome sequencing identifies expression patterns and genes, aiding understanding of genes, pathways, networks, and regulatory mechanisms. Third-generation sequencing, exemplified by PacBio Single Molecule Real-Time (SMRT) sequencing, uses ultra-long reads to directly sequence full-length cDNAs without fragmentation, obtaining high-quality full-length transcripts and overcoming NGS limitations (Abdel-Ghany et al., 2016; Rhoads and Au, 2015). Sequencing generates numerous reads for gene annotation, discovery, expression analysis, and regulatory pattern identification. Rosa-Santos et al. studied sugarcane’s aluminum stress response via RNA-Seq (Rosa-Santos et al., 2020). Raju et al. performed high-throughput sequencing on heat-stressed sugarcane, finding phytepsin, ferredoxin-dependent glutamate synthase, and DDR-48 expression increased threefold (Raju et al., 2020). Selvi et al. compared drought-responsive transcriptomes of sugarcane genotypes with varying tolerance, identifying key metabolic pathways and genes in water deficit alleviation via KEGG (Selvi et al., 2020). Ali et al. (2021) analyzed MAP kinase gene expression in sugarcane under biotic/abiotic stresses, showing distinct profiles under biotic (*Acidovorax avenae* subsp. *avenae, Xanthomonas albilineans*), abiotic (drought, salt), and SA treatments (Ali et al., 2021). Transcriptome sequencing is a common, effective method for studying stress resistance, identifying TFs, their targets, and regulatory networks in drought responses. Major TF families include AP2/EREBP (AP2/ERF), ABI3VP1, ARF, bZIP/HD-ZIP, C2H2, GRAS, MYB/MYC, zinc finger, MADS, NAC, and WRKY. Among these, bZIP (AREB/ABF), DREB (AP2/EREBP), MYB/MYC, NAC, and WRKY are linked to drought tolerance (Kumar et al., 2023). Ethylene response factors (ERFs), downstream of EIN3 pathways, have a conserved DNA-binding domain (Krannich et al., 2015). ERF1, part of JA and ethylene signaling, is highly induced under drought and salt stress (Hussain et al., 2021).

WGCNA identifies gene modules, analyzes module-phenotype relationships, and maps intra-module regulatory networks (Langfelder and Horvath, 2008). Lv et al. identified three modules and 12 key drought resistance genes via WGCNA (Lv et al., 2020). Wu et al. used WGCNA to reveal transcriptome dynamics in smut-infected sugarcane, aiding smut resistance breeding (Wu et al., 2022). WGCNA effectively identifies phenotype-associated modules/genes, aiding stress resistance research.

In this study, PEG6000 was used to simulate drought stress to identify stress-responsive genes and transcriptional regulators. We also analyzed how new sugarcane varieties respond to drought, providing a basis for drought-resistant breeding. To leverage their strengths while overcoming their limitation, PacBio was used to generate full-length transcriptome data and the deep-coverage data by Illumina was used to correct the full-length transcripts, which were then used as a reference. Via GO and KEGG enrichment, we identified drought-responsive DEGs and pathways. Drought-related TFs, key modules, and hub genes were screened by transcriptional analysis and WGCNA and validated by PCR.

## 2 Materials and methods

### 2.1 Acquisition and processing of sugarcane samples

The experimental materials used in this study were ZZ2, ZZ9, and ROC22 (control), sourced from the Fusui Sugarcane Planting Experimental Base of Guangxi University (Guangxi China-ASEAN Youth Industrial Park, China). ZZ2 and ZZ9 are new varieties bred in recent years by our laboratory (State Key Laboratory for the Protection and Utilization of Subtropical Agricultural Biological Resources) and share the same parent (ROC25 × Yunze 89-7), whereas ROC22 has been widely cultivated in Guangxi (Nanning City, China) over the past decade.

Seed stems with uniform bud development were selected for bucket cultivation in sand medium. Four groups were established: one control group (CK) and three drought-stress treatments for 1, 3, and 5 days (d), respectively. Sampling was conducted at specific time points, namely 5, 3, and 1 d before the termination of the drought treatment. Each group included three biological replicates (n=3). When sugarcane seedlings reached the 4–6 leaf stage, drought stress was simulated using PEG6000. Following the treatment, photosynthetic parameters and physiological indices were measured. Concurrently, leaf samples (n=3 biological replicates per group) were collected, immediately flash-frozen in liquid nitrogen, and stored at -80°C for subsequent RNA extraction.

### 2.2 RNA extraction and transcriptome sequencing

Thirty-six samples (from three sugarcane varieties, with four treatment groups and three biological replicates per variety) were used for second-generation transcriptome sequencing. For third-generation sequencing, mixed samples of the three varieties (ZZ9, ZZ2, and ROC22) under different stress treatments were used.

Total RNA was extracted from sugarcane samples using the TRIzol reagent method. RNA integrity (RIN value and 28S/18S ratio) was assessed using an Agilent 2100 Bioanalyzer. Qualified RNA was reverse-transcribed into cDNA using the SMARTer^®^ PCR cDNA Synthesis Kit. PCR amplification was performed using KAPA HiFi PCR Kits to construct a Sequel library with insert sizes of 0.5–6 kb. Full-length cDNAs were sequenced in real-time using the third-generation PacBio Sequel platform.

PacBio Sequel offline data were processed using HQRF (High-Quality Region Finder) to identify the longest region of singly loaded enzyme activity, and low-quality regions were filtered based on SNR (Signal-to-Noise Ratio). Quality values in the results of subsequent analyses were reliable after CCS and Arrow correction. After adapter trimming and low-quality read filtering, post-filter polymerase reads were obtained, followed by insert size quality control and transcript classification. After filtering with Lima, full-length polyA-containing transcripts were obtained. These transcripts were then clustered and corrected to generate high-quality consensus sequences, which were further corrected using second-generation sequencing data via LoRDEC (Zhao et al., 2024).

### 2.3 Transcriptome data analysis

Raw sequencing data were converted to sequence data and stored in FASTQ format. Base composition bias and sequencing quality were evaluated. Full-length transcripts were clustered and merged using cd-hit-est, followed by evaluation using N50, ExN50, and BUSCO (Simão et al., 2015) metrics. Short reads were aligned to the transcriptome reference using Bowtie (Langmead and Salzberg, 2012).

Sequence alignment and functional annotation were performed using BLAST (Altschul et al., 1997) and DIAMOND (Buchfink et al., 2015), with gene annotations obtained from seven databases (UniProt, KEGG, GO, Nr, PFAM, eggNOG, and KEGG Pathway). Gene expression levels were quantified using RSEM (Li and Dewey, 2011), and differential expression analysis was conducted using DESeq2 (Anders and Huber, 2010). Differentially expressed genes were subjected to GO and KEGG enrichment analyses.

### 2.4 Construction of WGCNA collaborative expression network

We performed WGCNA using the WGCNA R package (v1.68). Genes were pre-filtered by retaining the top 75% based on median absolute deviation (MAD), applying a threshold of MAD > 0.01. The analysis was conducted using a standardized gene expression matrix and corresponding trait data, with all other parameters set to their default values. Following identification of core modules, Gene Ontology (GO; Young et al., 2010) and KEGG Pathway (Xie et al., 2011) enrichment analyses were performed. Results were visualized using Cytoscape (Shannon et al., 2003) to screen for hub genes.

### 2.5 qRT-PCR validation

To validate the DEGs from RNA-seq, RT-qPCR analysis of six key genes was conducted, including hub genes strongly associated with drought-resistant traits that were screened from differential expression and WGCNA co-expression network analyses. Total RNA was reverse-transcribed into cDNA using the SPARK Script II RT Plus Kit (with gDNA Eraser) following the manufacturer’s instructions. qRT-PCR was conducted on a LightCycler480 system using TB Green^®^ Premix Ex Taq™ II (Tli RNaseH Plus), following the manufacturer’s protocol. Glyceraldehyde-3-phosphate dehydrogenase (GAPDH) served as the internal reference gene, with primer sequences listed in **Table 1**. Relative gene expression levels were calculated using the 2^-△△Ct^ method.

**Table 1:** Primers for RT-qPCR.

Gnen ID	Forward sequence (5’-3’)	Reverse sequence (5’-3’)
Unigene22830	TCGGCGTGCAAGAAAACTAGA	CCGACGAGACCCACCATCTC
Unigene14837	TGGTTCGGAAGCCATTGGTG	CGTATAGCGCGATCCCCCAAG
Unigene33688	GGGATAAGGTGCTGGTGGTGA	TCCGACGATTACATCAAGTTGGGA
Unigene21956	GACTTCCCCGAGATCGTGTGTT	TTCCCTGGCTCCTGGTCAAA
Unigene32430	AAGGCGCTCGTCTTCTACG	AGCCCGCCCTTCTTGTTG
Unigene4368	GCCTCAGGTTGGATGACTGG	CCACGAACGGCTTCTGCT
GAPDH	CACGGCCACTGGAAGCA	TCCTCAGGGTTCCTGATGCC

## 3 Results

### 3.1 Establishment of transcriptome database based on SMRT and illumina technology

To investigate the drought resistance mechanisms in sugarcane (ZZ9, ZZ2, and ROC22), building on previous studies, we used Illumina and PacBio platforms to conduct *de novo* transcriptome assembly and single-molecule real-time (SMRT) sequencing, aiming to obtain a comprehensive sugarcane transcriptome. The library generated 24.87–27.81 Gb of subreads. Full-length transcripts were obtained, followed by clustering and correction using Illumina data. Sequenced reads were filtered to retain high-quality data (valid data). Clustering and assembling these transcripts yielded a total sequence length of 77,930,985 bp.

Given that sugarcane is a heteropolyploid with a complex genome, and that hybrid offspring exhibit significant genomic variation, we performed *de novo* transcriptome analysis using transcripts from Illumina and PacBio sequencing as the reference transcriptome. Although this approach reduces comparative efficiency, it does not affect subsequent analyses.

Sequence statistics for all unigenes are presented in **Table 2**: 40,345 sequences with an average GC content of 51.75%. BUSCO (Benchmarking Universal Single-Copy Orthologs; http://busco.ezlab.org/) assesses genome/transcriptome assembly and annotation completeness using single-copy orthologs (Simão et al., 2015). Generally, a transcriptome is considered complete if >80% of complete BUSCOs are detected. In the absence of a sugarcane reference genome, complete BUSCOs accounted for 73% (**Table 3**), indicating that the obtained unigene sequences have good integrity.

**Table 2:** Unigenes sequential statistical results.

Item	Value
Total_length(bp)	77930985
Total_number	40345
GC_content (%)	51.75
N50(bp)	2112
N90(bp)	1302
Average(bp)	1931.61
Median(bp)	1858
Min(bp)	200
Max(bp)	7501

Total_Length, total length of all sequences; Total_Number, total number of sequences; GC_Content, average GC content of sequences; N50, length of the sequence at which the cumulative length reaches 50% of the total length after sorting sequences in descending order of length; N90, length of the sequence at which the cumulative length reaches 90% of the total length after sorting sequences in descending order of length; Average, average length of sequences; Median, median length of sequences; Min, minimum length of sequences; Max, maximum length of sequences.

**Table 3:** Integrity evaluation of unigenes.

Item	Number	Percent
Complete BUSCOs	313	73%
Complete and single-copy BUSCOs	103	24%
Complete and duplicated BUSCOs	210	49%
Fragmented BUSCOs	15	3.50%
Missing BUSCOs	101	23.50%
Total BUSCO groups searched	429	100%

Complete BUSCOs: detected complete BUSCOs. Integrity is defined as the average length of detected genes within the 95% confidence interval of BUSCO orthologous groups; Complete and single-copy BUSCOs: detected complete BUSCOs present as single copies; Complete and duplicated BUSCOs: detected complete BUSCOs present as multiple copies; Fragmented BUSCOs: fragmented (incomplete) detected BUSCOs; Missing BUSCOs: undetected BUSCO orthologous groups; Total BUSCO groups searched: number of BUSCOs in the database used.

Original image data from sequencing were converted to sequence data via base calling, referred to as raw data (raw reads). Data were stored in FASTQ format, containing sequence bases and sequencing quality scores. Low-quality reads and adapter sequences were filtered out, yielding valid data (clean data/clean reads). After filtering and quality control, Q30 values ranged from 92.29% to 94.90%, indicating no significant GC bias and good overall sequencing quality.

To obtain comprehensive gene functional information, SMRT sequencing transcriptomes and *de novo* assembled short-read transcriptomes were annotated via BLAST (http://blast.ncbi.nlm.nih.gov/Blast.cgi) by matching known proteins in UniProt, KEGG, GO, Nr, PFAM, eggNOG, and KEGG Pathway databases. Statistical results are shown in **Table 4**. The total number of unigenes was 40,359. Of these, 38,791 unigenes were annotated (96.11% of the total). The Nr database annotated the most unigenes (38,686, 95.85%) via BLAST, while KEGG Pathway annotation had the lowest proportion (26.25%). Notably, 1,568 unigenes remained unannotated, suggesting they may be novel genes. GO and KEGG annotated 30,480 and 15,900 unigenes, respectively.

**Table 4:** Statistical table of gene function annotation results of seven databases.

Item	Count	Percentage
All	40359	100%
Annotation	38791	96.11%
Uniprot	31138	77.15%
Pfam	33747	83.62%
GO	30480	75.52%
KEGG	15900	39.40%
Pathway	10594	26.25%
eggNOG	27394	67.88%
Nr	38686	95.85%

First column: “All” refers to all unigenes; “Annotations” refers to unigenes with at least one annotation; “KEGG” refers to unigenes annotated in the KEGG database; “UniProt” refers to unigenes annotated in the UniProt database; “eggNOG” refers to unigenes annotated in the eggNOG database; “GO” refers to unigenes annotated in the GO database; “Pathway” refers to unigenes annotated in the KEGG Pathway database; “Pfam” refers to unigenes annotated in the Pfam database; “Nr” refers to unigenes annotated in the Nr database. Second column: corresponding counts for each item; Third column: proportion of each item.

**Figure 1:**
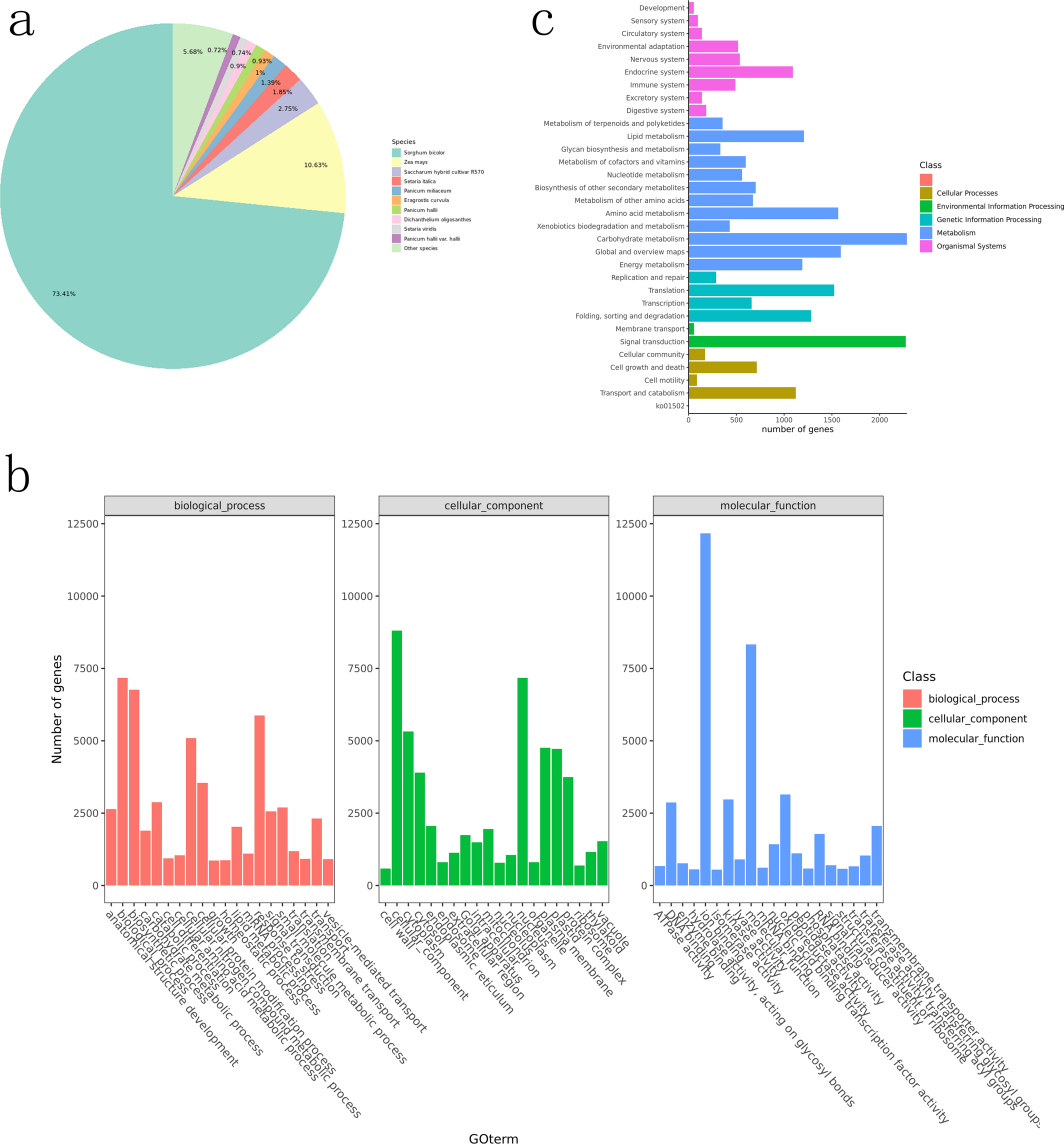
Functional annotation diagram. **(a)** statistics of Nr annotation results (species distribution map); **(b)** GO functional classification of unigenes; **(c)** KEGG Pathway classification.

In Nr annotations, BLAST comparisons showed the highest match with sorghum genomes (73.41%), followed by maize (10.63%) and Saccharum hybrid cultivar R570 (2.75%; **Figure 1a**), consistent with previous findings (Xu et al., 2018; Zheng et al., 2020).

GO (Gene Ontology) annotations were simplified to GOslim categories. Genes were categorized into three functional groups: cellular components, molecular functions, and biological processes. The top 20 most annotated GOslim terms in each category were selected for visualization (**Figure 1c**). In biological processes, >5,000 genes were annotated to biosynthetic processes, cellular nitrogen compound metabolic processes, and stress responses. In cellular components, cytoplasm and nucleus were the most abundant categories. Over 7,500 genes were enriched in molecular functions.

Genes annotated by KEGG were classified into metabolic pathways (**Figure 1b**), with the most enriched in metabolism. Notably, more genes were enriched in amino acid metabolism, carbohydrate metabolism, and global/overview maps. The highest number of genes in environmental information processing were enriched in signal transduction.

### 3.2 Analysis of differential gene expression under drought stress

To investigate changes in molecular mechanisms among and within sugarcane varieties under drought stress, we identified drought-responsive differentially expressed genes (DEGs) by analyzing gene expression levels. DEGs were identified from the raw count data using DESeq2, applying a threshold of an adjusted p-value (q-value) < 0.05 and |log2(fold change)| > 1. Interspecific comparisons were performed between ROC22 vs. ZZ2 and ROC22 vs. ZZ9; intraspecific comparisons were conducted between treatment groups (1d, 3d, 5d) and the control (CK) for each variety. DEG results are shown in **Table 5**. ZZ9, ROC22, and ZZ2 had 4,470, 5,036, and 7,577 DEGs, respectively, with ZZ2 showing the highest number among the three varieties. This variation may reflect differential impacts of drought stress across varieties, leading to varying changes in molecular mechanisms.

**Table 5:** Numbers of DEGs in each experimental-processing stage.

Sample	Up	Down	Total
ROC22-1_vs_ROC22-CK	1199	816	2015
ROC22-3_vs_ROC22-CK	1109	427	1536
ROC22-5_vs_ROC22-CK	934	551	1485
ZZ2-1_vs_ZZ2-CK	1518	684	2202
ZZ2-3_vs_ZZ2-CK	1265	1783	3048
ZZ2-5_vs_ZZ2-CK	1352	975	2327
ZZ9-1_vs_ZZ9-CK	1006	777	1783
ZZ9-3_vs_ZZ9-CK	518	483	1001
ZZ9-5_vs_ZZ9-CK	940	746	1686
ROC22-CK_vs_ZZ2-CK	5999	6271	12270
ROC22-CK_vs_ZZ9-CK	5091	5861	10952
ZZ2-CK_vs_ZZ9-CK	3059	3627	6686
ROC22-1_vs_ZZ2-1	5840	6500	12340
ROC22-1_vs_ZZ9- 1	5637	6176	11813
ZZ2-1_vs_ZZ9-1	3241	3374	6615
ROC22-3_vs_ ZZ2-3	6527	6065	12592
ROC22-3_vs_ZZ9- 3	5834	5861	11695
ZZ2-3_vs_ZZ9-3	4214	4976	9190
ROC22-5_vs_ZZ2-5	5249	5604	10853
ROC22-5_vs_ZZ9-5	4979	5335	10314
ZZ2-5_vs_ZZ9-5	3002	3359	6361

However, intervarietal comparisons under the same treatment showed relatively large numbers of DEGs per group, with no significant differences between groups. Moreover, ROC22_VS_ZZ2 had slightly more DEGs than ROC22_VS_ZZ9 in intervarietal comparisons, while ZZ2_VS_ZZ9 had the fewest DEGs among all intervarietal comparisons.

Intraspecific analysis showed ROC22–1 had the most DEGs (2,015), and ROC22–5 had the fewest (1,485). In ROC22-3, upregulated DEGs were more than twice the number of downregulated DEGs. ZZ2–3 had the most DEGs (3,048), while ZZ2–1 had the fewest (2,202). For ZZ9, ZZ9–3 had the fewest DEGs (1,001), and ZZ9–1 had the most (1,783).

Interestingly, except for ZZ2-3 (fewer upregulated than downregulated DEGs), all other treatment groups showed a higher proportion of upregulated than downregulated DEGs, with a higher proportion of upregulated DEGs after 1d treatment compared to 3d and 5d treatments. This suggests that as drought stress persists, fewer genes exert a promoting effect.

### 3.3 GO enrichment analysis

**Figure 2:**
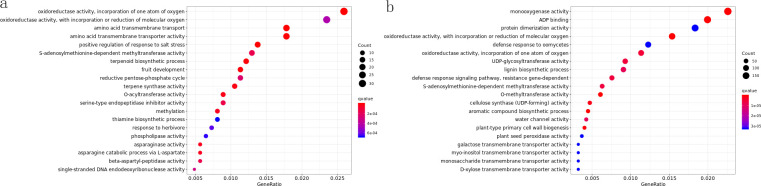
Go enrich dotplot. **(a)** ROC22-5_vs_ROC22-CK_GOenrich_dotplot; **(b)** ROC22-3_vs_Zz9-3_GOenrich_dotplot.

To clarify the molecular mechanisms underlying responses to the same treatment across varieties and different treatments within a variety, we performed intra- and inter-varietal GO enrichment analyses on DEGs. GO enrichment analysis for various gene sets was conducted using the GOseq R package, with an adjusted p-value (FDR) of < 0.05 defining statistically significant enrichment after correction for multiple testing. Gene Ontology (GO) annotates gene functions into three categories: cellular component (CC), molecular function (MF), and biological process (BP). For GO enrichment, the top 20 significantly enriched terms were selected for visualization. **Figure 2** shows one comparison group, with other charts provided in the appendix.

In GO analysis, compared with the control, all three varieties showed significantly enriched terms under 1d drought stress. In ZZ2-1, photosynthesis-related terms were the most enriched, whereas in ZZ2-3, monooxygenase activity was the top enriched term. In ZZ9, the most abundant term was “response to water deficit”.

In ROC22_vs_ZZ9, terms related to oxidative activity, ADP binding, terpene biosynthesis, jasmonic acid metabolism, and sugar alcohol transmembrane transporter activity were significantly enriched throughout drought stress. In ROC22_vs_ZZ2, however, sugar alcohol transmembrane transporter activity was significantly enriched only after 3d and 5d drought treatments.

Intraspecific comparisons revealed that all varieties significantly enriched multiple photosynthesis-related terms after 1d drought treatment. This suggests that drought stress induces responses in the photosynthetic system of all varieties to counteract external changes. After 3d drought stress, ROC22 enriched more photosynthesis- and oxidative activity-related terms, whereas ZZ2 and ZZ9 enriched more terms related to oxidative activity, plant hormones, polysaccharide synthesis, and flavonoid biosynthesis.

### 3.4 KEGG enrichment analysis

In organisms, genes coordinate to perform their biological functions. Pathway enrichment analysis identifies key biochemical metabolic and signal transduction pathways involving DEGs. KEGG (Kyoto Encyclopedia of Genes and Genomes) is the primary public pathway database (Xie et al., 2011). To further explore DEG involvement in metabolic pathways, we performed KEGG pathway enrichment analysis. KEGG pathway enrichment analysis for various gene sets was conducted using KOBAS (v2.0), with an adjusted p-value (FDR) of < 0.05 defining statistical significance after correction for multiple testing. The top 20 significantly enriched terms were selected for visualization. Significance was indicated by qvalue, defined as the pvalue corrected for multiple hypothesis testing. Qvalue ranges from 0 to 1, with smaller values indicating more significant enrichment.

KEGG enrichment results showed photosynthetic metabolic pathways were significantly enriched after 1d of drought stress. In ROC22, 19 terms were significantly enriched after 3d of stress, while the most enriched terms after 1d involved photosynthetic genes. In ZZ2, the most enriched term after 3d of stress was phenylpropanoid biosynthesis. In ZZ9, the most significant enrichment occurred after 3d, followed by 5d; after 5d, the most enriched genes mapped to the linoleic acid pathway.

Photosynthetic pathways and photosynthetic antenna proteins were more enriched after 1d, with reduced enrichment after 3d. For example, in ROC22-1_vs_ZZ2-1, pathways such as cutin, suberin, and wax biosynthesis; linoleic acid biosynthesis; ubiquinone and other terpene quinone biosynthesis; flavonoid biosynthesis; and cysteine/methionine metabolism were all downregulated. In contrast, photosynthesis-related genes were upregulated. Genes such as PRLY, PXG, LOX, TPS, PSBP, and PER were downregulated.

In ROC22-3_vs_ZZ2-3, most genes in pathways including flavonoid biosynthesis, arginine/proline metabolism, linoleic acid metabolism, pyruvate biosynthesis, alanine/aspartate/glutamate metabolism, and cutin/suberin/wax biosynthesis were upregulated. PER was downregulated, whereas LOX, PXG, and GUST were mostly upregulated. In ROC22-5_vs_ZZ2-5, TPS was downregulated.

**Figure 3:**
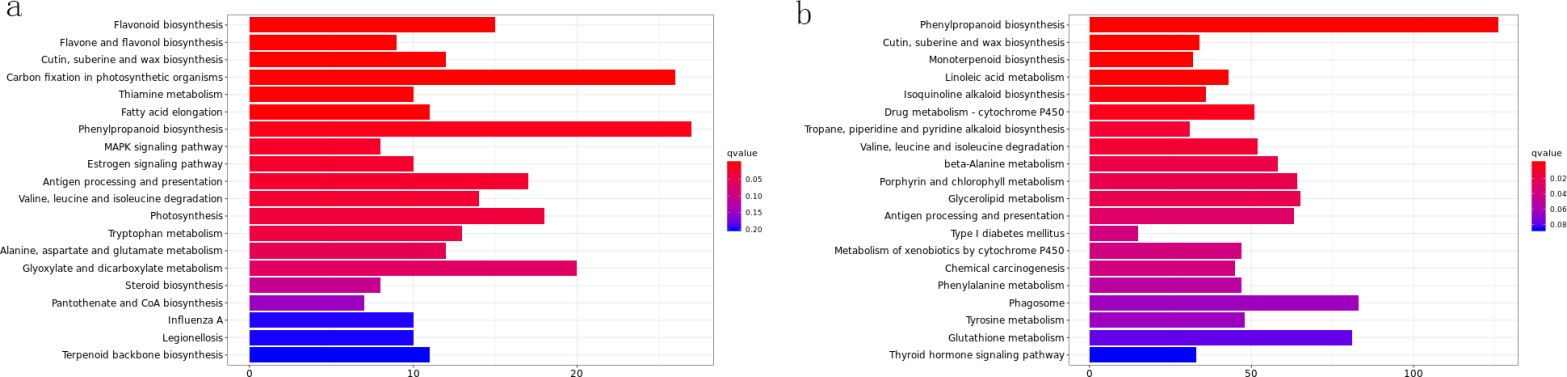
KEGGenrich_barplot. **(a)** ROC22-5_vs_ROC22-CK_KOenrich_barplot; **(b)** ROC22-3_vs_Zz9-3_KOenrich_barplot.

In ROC22-1_vs_ZZ9-1, genes in linoleic acid biosynthesis, isoquinoline alkaloid biosynthesis, arginine/proline metabolism, tyrosine metabolism, starch/sucrose metabolism, and flavonoid biosynthesis (including PXG, PRLY, LOX, P5CS2, GSTU, TPS, and PER) were all downregulated. In **Figure 3**, ROC22-3_vs_ZZ9-3, linoleic acid metabolism was downregulated, with PXG, TPS, and LOX also downregulated. Tyrosine metabolism was downregulated in ROC22-5_vs_ZZ9-5.

Glutathione metabolism and most related genes were downregulated in ZZ2-1_vs_ZZ9-1. In ZZ2-3_vs_ZZ9-3, drought resistance-related pathways (cutin/suberin/wax biosynthesis, arginine/proline metabolism, stilbene/diarylheptane/gingerol biosynthesis, and flavonoid biosynthesis) were significantly downregulated, indicating upregulation in ZZ9-3. In ZZ2-5_vs_ZZ9-5, most genes in phenylalanine metabolism, cutin/suberin/wax biosynthesis, and flavonoid biosynthesis (including LOX3, PER, PXG, and TPS) were downregulated, suggesting these pathways and genes may be critical for drought responses.

### 3.5 Regulation of transcription factor expression under drought stress

Transcription factors, also called trans-acting factors, are proteins with specific structural domains that regulate gene expression. They regulate target gene transcription by binding to cis-acting elements in gene promoters and modulate the co-expression of multiple genes. Previous studies have conducted extensive research on transcription factors, facilitating our investigation of drought resistance mechanisms.

In this study, 51 transcription factor families were identified, containing 1,432 significantly differentially expressed transcription factors. A large number of transcription factors in families including bHLH, WRKY, bZIP, ERF, NAC, G2-like, MYB-related, MYB, GRAS, and TALE are drought-inducible. The top 10 families accounted for 58.03% of all transcription factors, with the bHLH family containing the most (151). Notably, the DBB family included two highly expressed transcription factors: BBX24 (Unigene10424, Unigene22238, Unigene33728, Unigene35711) and BBX25 (Unigene10037), with some exhibiting FPKM values up to 1000.

**Figure 4:**
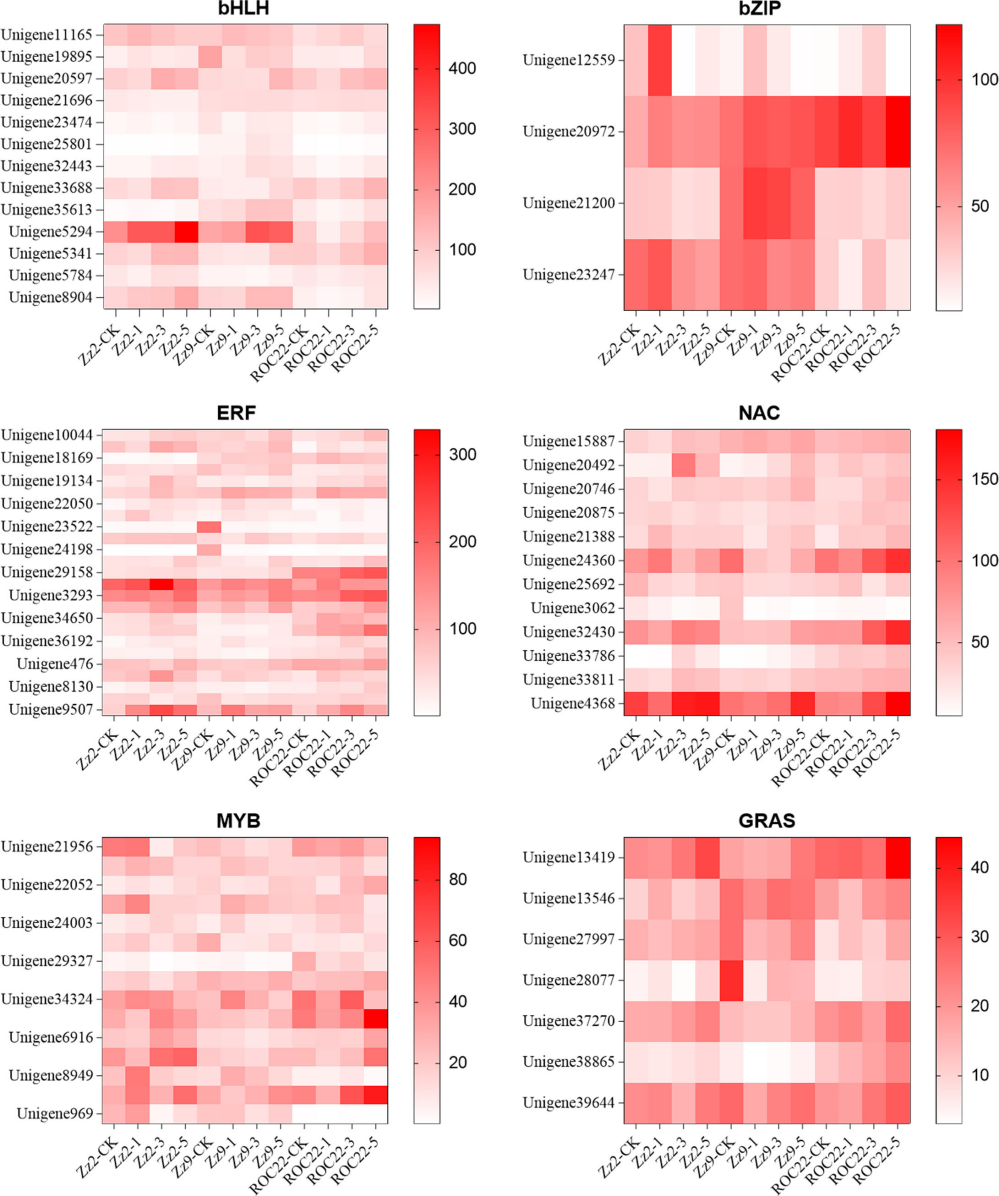
Expression heatmap of key transcription factor families in different sugarcane varieties under drought stress. Expression patterns (based on FPKM values) of selected genes from six key TF families (bHLH, bZIP, ERF, NAC, MYB, and GRAS) across three sugarcane varieties (ZZ9, ZZ2, and ROC22) under drought stress (0, 1, 3, and 5 days). Each row represents a gene, and each column represents a sample. The color scale indicates expression levels (red, high; blue, low). Both rows (genes) and columns (samples) were clustered by hierarchical clustering. Note the distinct TF expression profile, including the specific upregulation of a bZIP TF (Unigene21200) in variety ZZ9 under drought.

We selected the bHLH, bZIP, ERF, NAC, MYB-related, MYB, and GRAS families for cluster analysis. Each gene had at least one FPKM value >20 and a fold change >2. **Figure 4** shows that Unigene20597 (FPKM = 428), Unigene33688 (FPKM = 417), and Unigene5341 (FPKM = 433) are bHLH35 transcription factors. All three genes exhibited high expression in ROC22, suggesting they may play critical roles in drought stress; Unigene21200 (FPKM = 339) is not annotated as a bZIP, but exhibited higher expression in ZZ9 and lower expression in ZZ2 (FPKM = 114) and ROC22 (FPKM = 117), suggesting it may positively regulate drought stress responses.

The ERF family belongs to ethylene-responsive factors. Unigene20270, Unigene29158, Unigene29517, Unigene3293, and Unigene9507 were differentially expressed in the three varieties after drought stress. Among these, Unigene20270 (ethylene-responsive transcription factor 1) was upregulated in ZZ9 after drought treatment, upregulated in ROC22 after 1d of drought stress, and downregulated after 3d and 5d.

Among NAC family transcription factors, Unigene24360 is NAC67, and Unigene32430 and Unigene4368 are NAC48. MYB family transcription factors, including Unigene21956 (MYB20), Unigene29896 (MYB73), Unigene34324 (MYBAS2), MYB1 (Unigene34972, Unigene7890), MYB08 (Unigene6916), MYBc (Unigene8949), and MYB4 (Unigene9662), exhibited significant expression differences during drought treatment. Unigene21956 exhibited higher expression in ZZ2 under control conditions and 1d drought stress. Unigene21956, highly expressed in ZZ9 under control conditions, was downregulated after drought treatment, indicating a negative regulatory role in drought stress. Unigene29896, Unigene34324, and Unigene9662 exhibited low expression in ZZ2 but relatively high expression in ZZ9 and ROC22. These are all positive regulators. Unigene7890 exhibited higher expression only in ZZ2, suggesting it may negatively regulate drought stress responses.

Among GRAS family transcription factors, Unigene13419 and Unigene37270 exhibited lower expression in ZZ9 but higher expression in ZZ2 and ROC22. In ZZ9, their expression gradually increased with drought duration, suggesting they positively regulate drought stress responses. In contrast, Unigene13546 exhibited higher expression in ZZ9 (FPKM = 100) than in ZZ2 (FPKM = 51) and ROC22 (FPKM = 75); its expression was further upregulated after drought treatment compared with the control, indicating it is a positive regulator.

### 3.6 WGCNA analysis

#### 3.6.1 Co-expression network analysis of phenotypic related DEG

WGCNA is a widely used co-expression analysis method. In this study, WGCNA combined with dimensionality reduction converted tens of thousands of gene expression profiles into co-expression modules, enabling detailed analysis of module genes to identify target phenotype-related genes.

**Figure 5:**
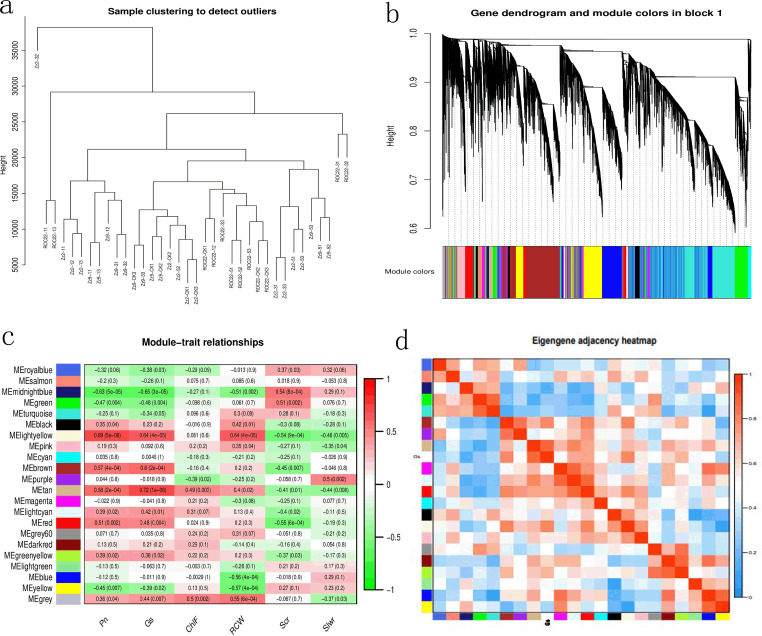
Correlation analysis between sample phenotypes and modules. **(a)** Sample clustering results; **(b)** Gene cluster diagram of Block 1, showing module correspondence; **(c)** Statistical results of module-trait correlations; **(d)** Heatmap of Gs-module correlations.

To investigate the relationship between gene expression and target trait phenotypes in the three sugarcane varieties under drought stress, we performed WGCNA. Cluster analysis results (**Figure 5a**) showed that most samples clustered together, with only ZZ2–32 deviating from the main group. Subsequent weighted co-expression analysis removed outliers based on distance. Two block diagrams were generated (Block 2 is provided in the appendix), with 22 modules in total, illustrating the correspondence between clustered genes and modules.

Correlation analysis revealed the strongest correlation (0.72) between the Metan module and Gs, followed by MEightyellow with Pn, MEightyellow with Gs, MEightyellow with RCW, and MEbrown with Gs, all with correlations >0.6.

Enrichment analysis of the target tan module identified 434 genes, including one gene each for NACA1, ABA, ERA1, PER70, and ATX; two SOD genes (SODF1, SODF2); two late embryogenesis abundant (LEA) protein genes; two LOX genes; four TSS genes; and four NRT1/PTR genes.

Subsequently, GO and KEGG enrichment analyses were performed. In **Table 6**. GO enrichment included 360 genes, with the most abundant terms being ribosome (30 genes), rRNA binding (21 genes), and thylakoid (21 genes). Forty-two GO terms were significantly enriched (p<0.05), with photosynthesis-related terms being the most abundant. Other significantly enriched terms included NAD(P)H dehydrogenase complex (plastid quinone), photosynthetic electron transport in photosystem I, photosynthesis, photosystem II components, glycolysis, fructose diphosphate aldolase activity, fructose 1,6-diphosphate metabolism, and auxin biosynthesis.

**Table 6:** Gene ontology enrichment analysis of tan module gene.

ID	Description	GeneRatio	BgRatio	pvalue	qvalue
GO:0005840	ribosome	30/360	278/30480	4.89130435902671e-20	2.18821510798563e-17
GO:0010598	NAD(P)H dehydrogenase complex (plastoquinone)	13/360	43/30480	1.8636994187275e-15	4.16880133136415e-13
GO:0019843	rRNA binding	21/360	233/30480	7.608054344304e-13	1.13453441976463e-10
GO:0009579	thylakoid	21/360	247/30480	2.35471611805259e-12	2.63356407940093e-10
GO:0009543	chloroplast thylakoid lumen	16/360	136/30480	7.45097165064294e-12	6.6666588453121e-10
GO:0009773	photosynthetic electron transport in photosystem I	12/360	65/30480	1.41003584206931e-11	1.05134251382361e-09
GO:0009534	chloroplast thylakoid	20/360	248/30480	2.07600519759233e-11	1.32677023906277e-09
GO:0031977	thylakoid lumen	12/360	79/30480	1.54598888426102e-10	8.64533257645966e-09
GO:0009658	chloroplast organization	16/360	261/30480	1.0419862536651e-07	5.17946383400783e-06

In **Table 7**, KEGG enrichment included 122 genes across 94 metabolic pathways. Notably, carbon fixation in photosynthetic organisms, fructose and mannose metabolism, aminoacyl-tRNA biosynthesis, and glycolysis/gluconeogenesis were significantly enriched.

**Table 7:** Enrichment analysis of the tan module gene KEGG pathway.

ID	Description	GeneRatio	BgRatio	pvalue	qvalue
ko00710	Carbon fixation in photosynthetic organisms	13/122	231/10594	2.41229290658387e-06	0.00021213115843438
ko00970	Aminoacyl-tRNA biosynthesis	11/122	173/10594	4.68661861657352e-06	0.00021213115843438
ko00051	Fructose and mannose metabolism	9/122	133/10594	2.17479806380186e-05	0.00065625485434021
ko00010	Glycolysis/Gluconeogenesis	12/122	290/10594	0.000124836228324886	0.00282524095682637
ko00030	Pentose phosphate pathway	8/122	151/10594	0.000345807842199192	0.00626094198508011
ko00906	Carotenoid biosynthesis	5/122	68/10594	0.00108984600578558	0.0164432906136069
ko00680	Methane metabolism	7/122	157/10594	0.00221624668804022	0.0286612353641291
ko03070	Bacterial secretion system	2/122	29/10594	0.0436244466169472	0.49364505382335
ko00780	Biotin metabolism	2/122	32/10594	0.0521265095100186	0.496041511763851

**Figure 6:**
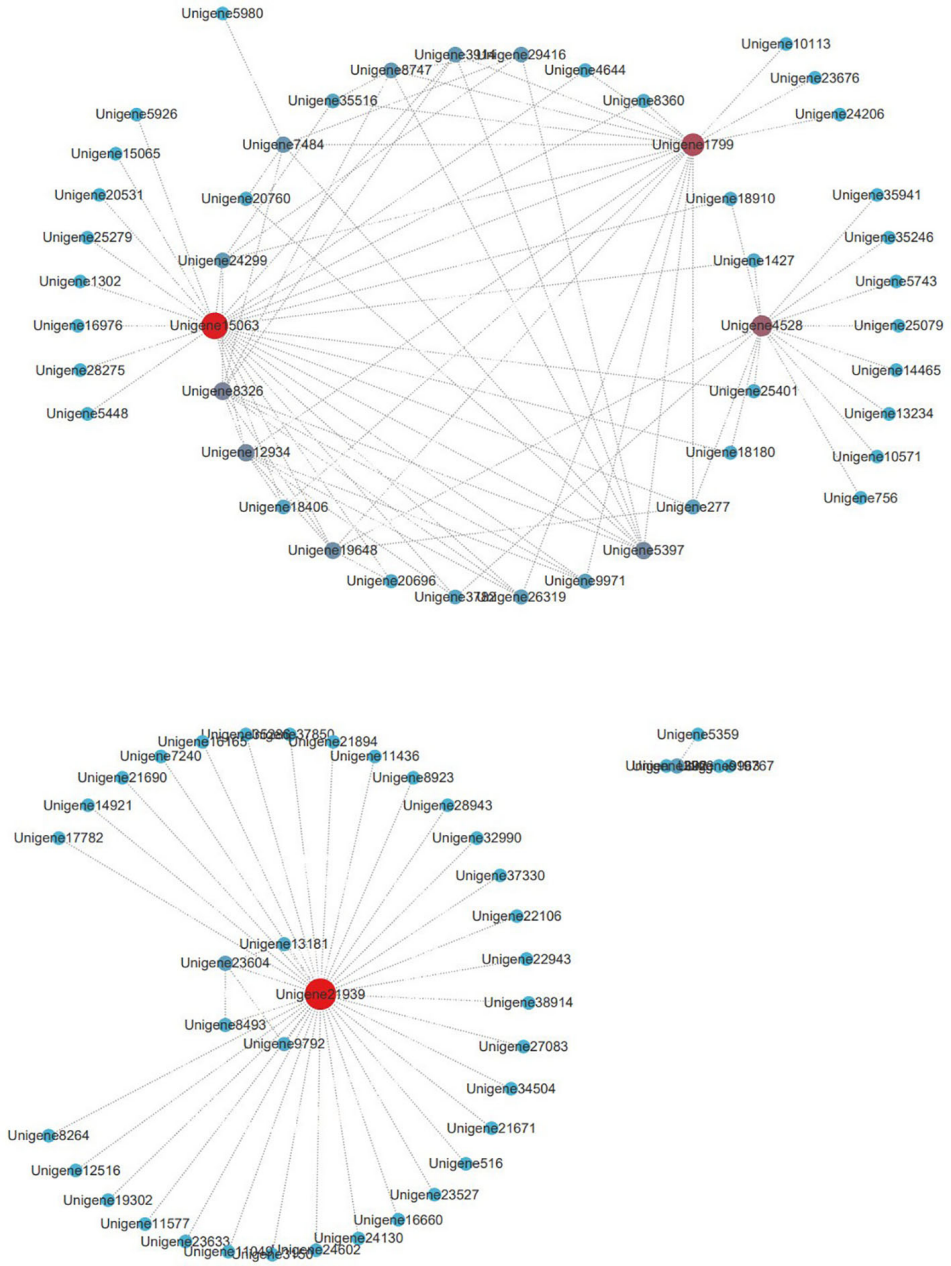
Gene network diagram of tan module.

Gene co-expression weights in the tan module were calculated, and genes with weights >0.25 were selected for visualization and analysis. Cytoscape visualization was performed for 132 genes in the tan module (**Figure 6**). Four hub genes were identified: Unigene21939, Unigene15063, Unigene1799, and Unigene4528, which play key regulatory roles in the tan module.

Unigene21939 is an unannotated novel gene that connects 35 genes, acting as a core gene within this subset. This suggests it may play an important role in drought stress responses. Unigene15063 encodes phytoene synthase 1 (PSY1), a key regulatory enzyme in carotenoid biosynthesis (Zhai et al., 2016) that connects 26 genes. Unigene1799 is annotated as an unspecified lipoprotein (syc1174_c) and connects 18 genes. Unigene4528 is a fibrin 5 homolog and connects 15 genes.

The roles of Unigene1799 and Unigene4528 in drought stress remain unclear; however, they represent potential candidate genes warranting further investigation.

### 3.7 qRT-PCR Validation of RNA-Seq Data

**Figure 7:**
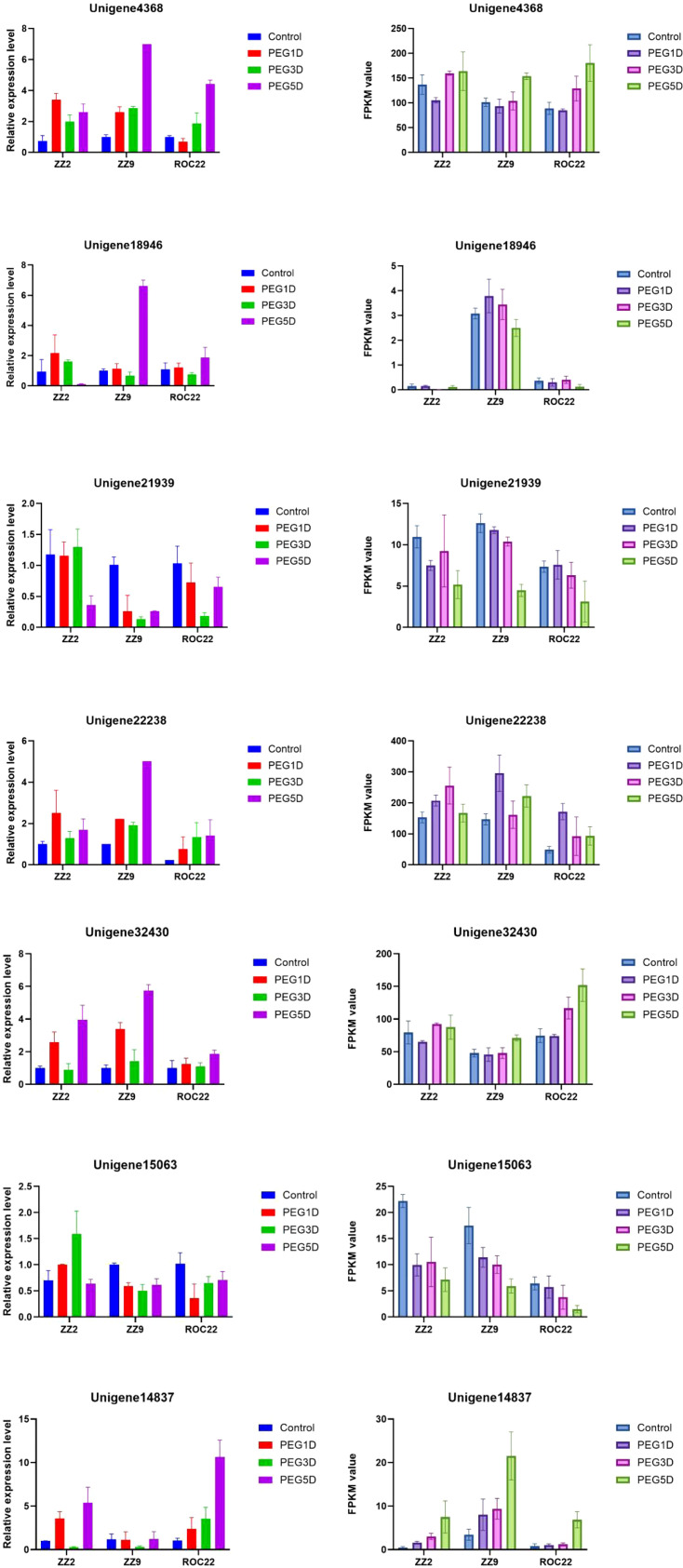
Real-time quantitative PCR and RNA-seq analysis of 6 genes.

To validate the accuracy of RNA-seq results, six DEGs were selected for qRT-PCR analysis (**Figure 7**). qRT-PCR results confirmed the RNA-seq data, as all six DEGs exhibited consistent expression trends, validating the reliability of the RNA-seq data.

## 4 Discussion

### 4.1 Differential gene expression in different sugarcane varieties

High-throughput sequencing, an efficient, reliable, and cost-effective transcriptome analysis technology, is widely used in non-model organisms. Sugarcane, a non-model plant with high polyploidy, has a complex genome, leading to significant genotypic differences among varieties. RNA sequencing is a powerful tool for deciphering transcriptomes, enabling gene annotation, discovery, expression analysis, and identification of biological regulatory patterns through large-scale read acquisition. Illumina and single-molecule real-time (SMRT) RNA sequencing technologies offer distinct advantages for investigating drought resistance mechanisms in sugarcane. This study is the first to integrate second-generation sequencing (Illumina) and SMRT sequencing for comprehensive transcriptome analysis, thoroughly investigating the molecular response mechanisms of different sugarcane varieties under drought stress.

SMRT sequencing generated a transcriptome with a total length of 68,485,627 bp, which was corrected using Illumina data to a final length of 77,930,985 bp. In total, 40,359 unigenes were identified (N50 = 2,112 bp). Annotation against seven major databases yielded 38,791 annotated unigenes, with 30,480 and 15,900 annotated by GO and KEGG, respectively. The Nr database showed the highest proportion of matches with sorghum. This sequencing generated relatively complete sugarcane transcriptome data, laying a foundation for subsequent research on drought resistance mechanisms and providing a reference for breeding drought-resistant varieties (Wang et al., 2020).

For drought resistance-related DEG analysis, intraspecific and interspecific comparisons were performed. Intraspecific comparisons revealed more upregulated than downregulated DEGs, with a higher proportion of upregulated DEGs at 1d than at 3d and 5d of drought stress. This may reflect an initial, broad activation of positive regulatory genes in early drought stages to counteract external stress (Contiliani et al., 2022; Valarmathi et al., 2023). A more nuanced interpretation of the attenuated upregulation during prolonged drought involves considering tissue-specific dynamics (Riccio-Rengifo et al., 2024). The whole-shoot transcriptome is a composite signal, and its temporal shift may arise from an increasing dichotomy between tissues. For instance, sustained upregulation in stress-signaling centers (e.g., vasculature) could be offset by widespread downregulation in tissues prioritizing energy conservation (e.g., leaf mesophyll) at 3d and 5d (Valarmathi et al., 2023; Min et al., 2020). This spatial divergence in transcriptional programming means the overall profile change reflects a complex tissue-level reallocation of function rather than a uniform reduction in transcriptional promotion. Among the three varieties, ZZ9 had the fewest DEGs, ZZ2 the most; ROC22_VS_ZZ2 had slightly more DEGs than ROC22_VS_ZZ9, while ZZ2_VS_ZZ9 had the fewest DEGs across all interspecific comparisons. ZZ2 and ZZ9 share a common parent, distinct from ROC22’s parent, explaining their minimal differences.

GO enrichment analysis of DEGs showed significant enrichment in ROC22_vs_ZZ9 for drought-responsive terms related to oxidative activity, ADP binding, terpene biosynthesis, jasmonic acid metabolism, and sugar alcohol transmembrane transporter activity. In ROC22_vs_ZZ2, sugar alcohol transmembrane transporter activity was significantly enriched only at 3d and 5d of drought treatment. This suggests ZZ9 better deploys coping strategies to mitigate external environmental damage under stress.

Intraspecific comparisons showed significant enrichment of multiple photosynthesis-related terms in all varieties at 1d of drought stress, indicating that drought stress triggers photosynthetic system responses to counteract external changes. At 3d of drought stress, ROC22 enriched more photosynthesis- and oxidative activity-related terms, while ZZ2 and ZZ9 enriched more terms related to oxidative activity, plant hormones, polysaccharide synthesis, and flavonoid biosynthesis.

KEGG enrichment analysis revealed numerous photosynthesis-related pathways in early drought stages, highlighting photosynthesis’s key regulatory role in early stress responses. In ROC22_VS_ZZ2, photosynthesis was upregulated at 1d of stress, suggesting ROC22 copes with early drought damage via photosynthesis. Under the same conditions, ZZ2 activates deeper mechanisms to resist damage, including biosynthesis of cutin, suberin, and wax; linoleic acid, ubiquinone, and other terpene quinones; flavonoids; cysteine and methionine metabolism; and genes such as PRLY, PXG, LOX, TPS, PSBP, and PER. At 3d of stress, most genes in pathways such as flavonoid biosynthesis, arginine/proline metabolism, linoleic acid metabolism, pyruvate biosynthesis, alanine/aspartate/glutamate metabolism, and cutin/suberin/wax biosynthesis were upregulated, along with LOX, PXG, and GUST. This suggests that as stress intensifies, ROC22 activates additional drought resistance-related metabolic pathways.

In ROC22-1_vs_ZZ9-1, pathways including linoleic acid biosynthesis, isoquinoline alkaloid biosynthesis, arginine/proline metabolism, tyrosine metabolism, starch/sucrose metabolism, and flavonoid biosynthesis were downregulated, along with genes such as PXG, PRLY, LOX, P5CS2, GSTU, TPS, and PER. The differential expression of these pathways and genes may be associated with post-stress resistance. In ROC22-3_vs_ZZ9-3, linoleic acid metabolism was downregulated, with PXG, TPS, and LOX also downregulated; tyrosine metabolism was downregulated in ROC22-5_vs_ZZ9-5. This indicates these pathways play a positive regulatory role in ZZ9 under stress, with the same pattern in ZZ2_vs_ZZ9, suggesting ZZ9 is more stress-tolerant than the other two varieties under external environmental pressure.

Transcription factors, as molecular switches regulating stress-responsive gene expression, play critical roles in abiotic stress responses, including regulatory networks involving bZIP, NAC, AP2/ERF, and MYB families (Martin et al., 2021). As trans-regulatory elements, they bind to specific cis-regulatory elements in target gene promoters to activate or repress target gene expression (Guo and Gan, 2006), critical for stress resistance and response.

This study identified 51 transcription factor families containing 1,432 significantly differentially expressed transcription factors. Numerous transcription factors in families including bHLH, WRKY, bZIP, ERF, NAC, G2-like, MYB-related, MYB, GRAS, TALE, and DBB are drought-inducible. Studies have shown water deficit induces ERF upregulation in sugarcane leaves (Wang et al., 2019); ERFs are ethylene-responsive factors. Five ERF genes were differentially expressed across the three varieties after drought stress; Unigene20270 (ethylene-responsive transcription factor 1) was upregulated in ZZ9 post-drought, suggesting it may function in ZZ9’s drought response.

Sharma et al. (2018) found that WRKY, NAC, MYB, AP2/ERF, and bZIP families are highly enriched in all gene sets, regulating 56% of common gene expression under drought and cold stress. Previous studies identified NAC as drought-inducible, with AhNAC2 a key participant in ABA signaling within the NAC family (Jia et al., 2019; Xiong et al., 2025; Borgohain et al., 2019). MYB transcription factors are characterized by a DNA-binding MYB domain; some (e.g., MYB20, MYB1, MYB4, MYBC, and MYB-related) regulate stomatal movement in drought responses and are reported to mediate abiotic stress in plants (Zhang et al., 2025; Zhu et al., 2023; Duan et al., 2023).

In this study, MYB20, MYB73, MYBAS2, MYB1, MYB08, MYBC, and MYB4 were significantly expressed under drought stress, suggesting they may be involved in drought regulation. MYBAS2 and MYB08, unreported in drought resistance, warrant further investigation. BHLH, WRKY, bZIP, G2-like, GRAS, and TALE were also significantly expressed. Functional analyses show bHLH transcription factors play key roles in biological processes including plant development, flavonoid biosynthesis, flowering, and photosynthesis (Khan et al., 2018). In this study, three bHLH35 genes were differentially expressed under drought stress, consistent with Dong et al. (2014).

Notably, Unigene21200 (a bZIP unannotated gene) exhibits higher expression in ZZ9 than in ZZ2 and ROC22, suggesting it may function as a novel drought-responsive gene. GRAS proteins are known to play important roles in abiotic stress responses (e.g., drought, salinity; Zhang et al., 2022). Wang et al. (2020) found GmGRAS37 is significantly upregulated under drought, salt, abscisic acid, and brassinosteroid treatments. This is consistent with differential GRAS gene expression in this study, with upregulation in all three varieties under drought stress.

Notably, two DBB family genes showed high expression and differential expression across varieties after drought stress. As a novel zinc finger transcription factor, DBB has unreported functions in abiotic stress, offering new directions for future research.

### 4.2 WGCNA analysis

Weighted gene co-expression network analysis (WGCNA) identifies candidate hub genes associated with specific functions or traits via hierarchical clustering of genes in co-expression networks (Langfelder and Horvath, 2008; Liang et al., 2020). Highly connected nodes in expression networks are defined as hub genes and are typically involved in biological processes and interactions. This study used dimensionality reduction to convert gene expression profiles into co-expression modules, followed by detailed analysis of module genes to identify target phenotype-related genes.

During this process, the Metan, MEightyellow, and MEbrown modules—showing strong correlation with Gs—were selected. MEightyellow also showed correlations >0.6 with Pn and RCW. Enrichment analysis of the well-correlated tan module identified 434 genes, including one gene each for NACA1, ABA, ERA1, and PER70; two SOD genes (SODF1, SODF2); two late embryogenesis abundant (LEA) protein genes; two LOX genes; four TSS genes; and four NRT1/PTR genes.

Studies have shown that HvSNAC1 is strongly induced under abiotic stresses (e.g., drought), and its overexpression improves water status, photosynthetic activity, and reduces water loss under drought (Kurowska and Daszkowska-Golec, 2023). This suggests NACA1 may participate in photosynthesis under drought and play a key role in drought resistance. Under drought stress, ABA activates defense mechanisms, regulates stomatal aperture, and induces defense-related gene expression, thereby alleviating environmental stress (Zareen et al., 2024). The ABA (abscisic acid) signaling pathway is central to regulating key factor expression and adaptive physiological responses to abiotic stress in plants (Danquah et al., 2014). Ogata et al. (2020) showed that CRISPR/Cas9-mediated OsERA1 mutation enhances rice responses to ABA and drought stress.

PER70 (a peroxidase gene) remains unreported, but peroxidases are enzymes critical for abiotic stress responses. Superoxide dismutases (SODs) are a family of metal enzymes involved in scavenging reactive oxygen species (ROS) (Zhou et al., 2019), enabling resistance to abiotic stresses. Late embryogenesis abundant (LEA) proteins are a large family of hydrophilic proteins critical for plant resistance to drought and other abiotic stresses (Magwanga et al., 2018). Lin et al. (2021) analyzed the roles of LEA proteins and abscisic acid-stress-ripening (ASR) genes in the rose superfamily, as well as their involvement in plant salt/alkaline tolerance and drought tolerance, via genome-wide analysis. Chen et al. (2021) found that SmLEA gene promoters contain multiple abiotic stress-responsive cis-acting elements, show tissue-specific expression, and most SmLEA genes are induced by drought stress.

In GO and KEGG enrichment analyses of the tan module, 360 genes were enriched in GO terms, with most enriched terms related to photosynthesis (e.g., NAD(P)H dehydrogenase complex [plastid quinone], photosynthetic electron transport in photosystem I, photosynthesis, and photosystem II components). Glycolysis, fructose diphosphate aldolase activity, and auxin biosynthesis were also significantly enriched. This indicates that photosynthesis-related pathways are activated after drought, and glycolysis—a respiratory fermentation pathway—serves as the primary mode of continuous ATP production to support energy metabolism (Khanna et al., 2014). Fructose diphosphate aldolase, a key enzyme in plant gluconeogenesis, glycolysis, and the Calvin cycle, is critical for plant growth and development. Zhao et al. (2021) found that tobacco NtFBA7/8 and NtFBA13/14 are important for photosynthesis and abiotic stress, respectively.

Auxin is mainly synthesized in plants via tryptophan-dependent pathways. LEA proteins, drought-induced products, have their expression directly regulated by IAA concentration; under drought, reduced auxin levels promote LEA accumulation, enhancing drought resistance (Shinozaki and Yamaguchi-Shinozaki, 2007). After drought stress, 122 genes and 94 metabolic pathways were enriched in KEGG, with significant enrichment in carbon fixation in photosynthetic organisms, fructose and mannose metabolism, aminoacyl-tRNA biosynthesis, glycolysis, and gluconeogenesis. Studies show drought reduces oligosaccharide accumulation (e.g., sucrose) but increases monosaccharide accumulation (e.g., mannose, inositol) in carbohydrate metabolism (Xiong and Ma, 2022), highlighting the importance of photosynthesis, glycolysis, gluconeogenesis, other carbohydrate metabolic processes, and plant hormone synthesis in drought responses.

## 5 Conclusion

This study delineates the transcriptomic landscape of sugarcane under drought stress by integrating second- and third-generation sequencing. Crucially, it reveals distinct drought-response strategies among the varieties ZZ9, ZZ2, and ROC22. The newly bred variety ZZ9 demonstrated a superior profile, characterized by a more targeted transcriptional reprogramming with fewer differentially expressed genes (DEGs) yet significant enrichment in core pathways like “response to water deficit.” This efficient response suggests a pre-adapted mechanism that minimizes metabolic cost while activating critical defenses, in contrast to the broader, less optimized reactions of ROC22 and ZZ2.

This resilience is further corroborated by WGCNA, which identified a key module highly correlated with stomatal conductance (Gs), enriched for genes involved in photosynthesis and ABA signaling. Hub genes within this module, along with variably expressed transcription factors, provide strong candidates for future validation.

In summary, our findings strongly indicate that ZZ9 possesses molecular traits conferring enhanced drought resilience compared to ROC22 and ZZ2. Its efficient transcriptomic response underscores its value as superior germplasm, and the identified candidate genes and pathways offer critical resources for breeding high-yielding, drought-tolerant sugarcane varieties.

## Data Availability

The datasets presented in this study can be found in online repositories. Transcriptome data reported in this paper has been deposited in Sequence Read Archive (SRA) repository, BioProject: PRJNA1305544.
